# The Role of Cytomorphometric Image Analysis in the Diagnosis of Thyroid Nodules

**DOI:** 10.7759/cureus.37872

**Published:** 2023-04-20

**Authors:** Jai K Chaurasia, Abeer M Ilyas, Vaishali Walke, Vikas Gupta, Neelkamal Kapoor

**Affiliations:** 1 Pathology and Laboratory Medicine, All India Institute of Medical Sciences, Bhopal, IND; 2 Otorhinolaryngology, All India Institute of Medical Sciences, Bhopal, IND

**Keywords:** parameters, image analysis, thyroid nodules, cytomorphometry, the bethesda system for reporting thyroid cytopathology-tbsrtc

## Abstract

Introduction

Fine needle aspiration cytology (FNAC) plays a vital role in the diagnosis of thyroid nodules. However, it is challenging due to the heterogeneity of thyroid nodules, overlapping cytomorphological features, and interobserver variability. Cytomorphometric analysis turns subjective observations into quantitative values. In this study, we performed cytomorphometric image analysis on cytological smears of thyroid nodules, classified according to The Bethesda System for Reporting Thyroid Cytopathology (TBSRTC).

Materials and methods

A retrospective analysis of Papanicolaou (PAP) and Hematoxylin & Eosin (H&E) stained fine needle aspirate smears from 50 patients with thyroid nodules with available follow-up histopathology was performed for a period of two years (March 2021 - March 2023), after obtaining approval from the institutional human ethical committee (IHEC-LOP/2020/IM0355). The nodules were categorized according to TBSRTC and were then subjected to cytomorphometric image analysis. Each nucleus was analyzed for 14 parameters, including aspect ratio, intensity, diameter, perimeter, roundness, area, fractal dimension, feret diameter, circularity, radii, fournier description, and chromatin texture parameters such as heterogeneity and clumpiness. The data obtained was analyzed through relevant statistical methods using SPSS version 23 (IBM Inc., Armonk, New York) and was compared by using the analysis of variance (ANOVA) test and post hoc test.

Results

Our results revealed that cytomorphometric image analysis not only distinguishes benign and malignant thyroid nodules but also can aid in categorizing thyroid nodules with predominant follicular patterns, such as follicular variant of papillary carcinoma, follicular adenoma and follicular carcinoma (p<0.001).

Conclusions

Morphometric analysis of cytological smears combined with cytomorphology has the potential to be an important tool in the diagnosis of thyroid nodules. It can improve diagnostic accuracy for better treatment and improved prognosis.

## Introduction

Fine needle aspiration cytology (FNAC) plays an important role in the diagnosis of thyroid nodules. The Bethesda System for Reporting Thyroid Cytopathology (TBSRTC) is a standardized, category-based tiered reporting system for FNAC of thyroid for reducing interobserver variability and enhancing reproducibility [1]. However, categorization of thyroid nodules is sometimes challenging, particularly in indeterminate category (category IV) follicular neoplasm/ suspicious of follicular neoplasm (FN/SFN) of the TBSRTC due to heterogeneity of thyroid nodules and overlapping cytomorphological features [2,3]. Moreover, misinterpretation and failure to recognize subtle morphological and architectural patterns of cells also pose diagnostic challenges. Nuclear cytomorphometric image analysis is an innovative technique that helps to provide a better objective analysis of nuclear features in the form of quantitative values. In this study, we have attempted cytomorphometric image analysis on FNAC smears of thyroid nodules for distinguishing not only benign and malignant thyroid nodules but also attempted to categorize thyroid nodules with predominant follicular patterns, such as follicular variant of papillary carcinoma, follicular adenoma, and follicular carcinoma based on cytomorphometry.

## Materials and methods

A retrospective analysis of Papanicolaou (PAP) and Hematoxylin & Eosin (H&E) stained fine needle aspirate (FNA) smears of 50 patients with thyroid nodules, with available follow-up histopathology, was done in the department of pathology and lab medicine for a period of two years (March 2021 - March 2023), after obtaining approval from the Institutional Human Ethical Committee (IHEC-LOP/2020/IM0355).

Selection method for cases

In this retrospective analysis of FNAC smears, only those FNAC smears of patients were retrieved and subjected to cytomorphometric analysis, where confirmatory follow-up histopathological diagnosis was available. First, a separate list of cases with benign and malignant confirmed histopathological diagnoses of thyroid nodules was prepared from the archives. Fifteen consecutive cases in each benign and malignant category (excluding follicular adenoma and follicular carcinoma) were selected. Corresponding FNAC slides of these cases were retrieved and examined and then categorized according to TBSRTC. FNAC smears not fulfilling the inclusion criteria (mentioned below) were excluded. Similarly, 20 consecutive cases with cytological diagnosis of follicular neoplasm/ suspicious of follicular neoplasm (FN/SFN) and with available histopathological confirmation (of follicular adenoma/ carcinoma) were selected (Table 1). FNAC smears with only cytological diagnosis of FN/SFN without histological confirmation and smears not fulfilling the inclusion criteria were excluded. Finally, FNAC smears from selected 15 cases from benign and malignant categories each and 20 cases from the FN/SFN category were subjected to cytomorphometric analysis (Table 1). 

**Table 1:** Distribution of the cases according to the TBSRTC into benign, malignant, and follicular neoplasm/suspicious of follicular neoplasm subjected to cytomorphometric image analysis with the corresponding histopathological diagnosis FVPC - follicular variant of papillary carcinoma, MNG - multinodular goitre, FA - follicular adenoma, FC - follicular carcinoma, PC - conventional papillary carcinoma, TBSRTC - The Bethesda System for Reporting Thyroid Cytopathology

	Distribution of cases according to the Bethesda system for reporting thyroid cytopathology					
	Benign (n=15)	Number of cases (percentage)	Follicular neoplasm/ suspicious of follicular neoplasm (FN/SFN) (n=10)	Number of cases (percentage)	Malignant (n=15)	Number of cases (percentage)
Histopathological diagnosis	Colloid nodule	7 (46%)	Follicular adenoma (FA)	11 (55%)	Conventional papillary carcinoma (PC) + follicular variant of papillary carcinoma (FVPC)	6 PC + 4 (FVPC) = 10 (66%)
	Multinodular goitre (MNG)	4 (26%)	Follicular carcinoma (FC)	9 (45%)	Medullary carcinoma	2 (13%)
	Hyperplastic/ adenomatoid Nodule	2 (13%)			Anaplastic carcinoma	2 (13%)
	Lymphocytic thyroiditis	2 (13%)			Poorly differentiated carcinoma	1 (6%)
	Total (N)	15 (100%)	Total	20 (100%)	Total	15 (100%)

Category one (non-diagnostic/ unsatisfactory) of TBSRTC was not included in this study as the aspirates in this category had insufficient diagnostic material (qualitative and/ or quantitative) to provide a conclusive opinion or interpretation. Follow-up histopathology was not available for obvious reasons. Similarly, category three (atypia of undetermined significance) of TBSRTC was also not included as confirmatory histology was not available in most cases as these cases were treated symptomatically with follow-up visits. Category five of TBSRTC (suspicious of malignancy) was also not included in this study as this category had cases with overall cytologic features of malignancy but couldn't be classified in the frank malignant category due to limited/ sparse cellularity. Cytomorphometry was not applied to this category because the malignant cells were often easily recognized, and the cytomorphometric analysis on this category nodules would have yielded results that would have been in concordance with the cytomorphometric results of the malignant category only. Moreover, the quantity/ number of frankly malignant cells was a limiting factor for getting statistically significant results.

Inclusion and exclusion criteria

Papanicolaou (PAP) and Hematoxylin & Eosin (H&E) stained FNAC smears with well-preserved nuclear morphology only were included. FNAC smears without histopathological confirmation were excluded. Also, smears showing extensive nuclear overlap with poor morphology, smears with obscuring inflammation, and blood and mucoid material were excluded. Smears showing cellular degenerative changes or drying artefacts were excluded.

**Figure 1:**
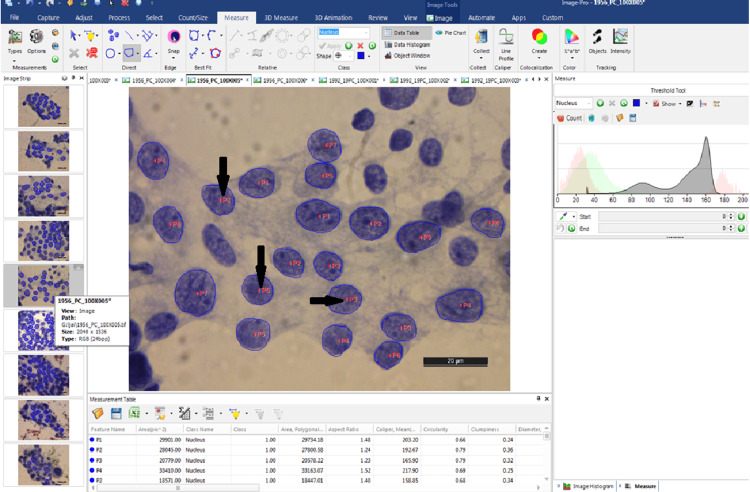
Software-developed image of thyroid epithelial cells in a case of papillary carcinoma of the thyroid, whereby each nucleus was labeled with a specific number (arrows)

The nucleus of each cell was first focussed at 1000 X (oil immersion) objective lens present on the Leica DFC295 camera system (Leica Microsystems, Wetzlar, Germany). Fifty nuclei of follicular cells per case with well-preserved morphology were selected. Nuclei showing overlapping, obscured nuclei with inflammation, blood or mucin, and nuclei showing degenerative changes were avoided. Isolated nucleus or nuclei just touching each other without overlap with a clean background were preferred and selected. The image of each nucleus was then captured through the attached digital camera and subsequently subjected to morphometric analysis through Image Pro Software (version 10; MediaCybernetics, Rockville, Maryland). This software labels each nucleus in the image with a specific number (Figure 1) and then quantifies its morphometric parameters.

Morphometric quantification of the following 14 parameters was done, as shown in Table 2.

**Table 2:** Morphometric parameters measured/ quantitated in the study

Parameters	Definition and description
Aspect ratio	Ratio of major axis and minor axis of the ellipse equivalent to the region.
Intensity (mean)	Calibrated image luminance at a point.
Diameter (mean)	Average length of diameters measured at numerous angles intervals and passing through region's centroid.
Perimeter (polygonal)	Length of the region’s boundary.
Roundness	Measure of roundness of nuclei. Calculated as Perimeter^ 2 ^/4x pix area
Area (polygonal)	Area of the polygon defining the region's outline.
Fractal dimension	It is a ratio providing a statistical index of complexity and represents measure of fractal pattern changes with the scale at which it is measured.
Feret diameter (Caliper mean)	Mean Diameter of array of numerous caliper lengths.
Margination	Relative distribution of region intensity between the center and the margin.
Heterogeneity	Fraction of pixels that deviate more than the Intensity range (10%) from the average intensity.
Clumpiness	Fraction of pixels deviating from the average, reflecting chromatin texture variation.
Circularity	Ratio of the area of an object against a circle whose diameter is equal to the objects's maximum Feret diameter.
Radii (mean)	Array of numerous angle radii.
Fournier description	Spectrum of the outline. Rotation invariant shape measurement.

Statistical analysis

The data obtained was analyzed with SPSS version 23 (IBM Inc., Armonk, New York) using standard statistical methods. The mean with standard deviation was calculated for each parameter in each category - benign, FN/SFN, and malignant of TBSRTC. The means for all morphometric parameters for these three categories were compared using the analysis of variance (ANOVA) test. A post hoc test (Games-Howell multiple comparison test) was also applied. A p-value of < 0.05 was considered significant. Similarly, to compare thyroid nodules with predominant follicular patterns, ANOVA and post hoc tests were also applied.

## Results

**Figure 2:**
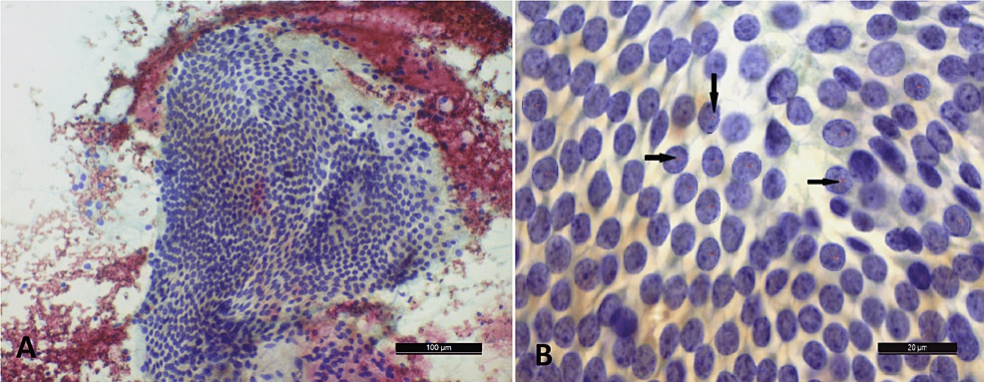
A) Sheet of benign follicular cells in a case of multinodular goiter (PAP x40). B) Corresponding software image with labeled nuclei (arrows) (x1000) PAP - Papanicolaou

In this retrospective analysis of thyroid nodules, the age of the patients ranged from 18 years to 70 years. The mean age for patients with benign follicular nodules was 36.40 years, while the mean age of patients with malignant nodules was 49.52 years. Among thyroid benign follicular nodules (N=15) (Table 1) subjected to cytomorphometric analysis, colloid nodule (CN) was the most common diagnosis (7; 46%), followed by multinodular goitre (MNG) (4; 26%) (Figure 2), hyperplastic /adenomatoid nodule (2; 13%) and lymphocytic thyroiditis (2; 13%) (Table 1).

**Figure 3:**
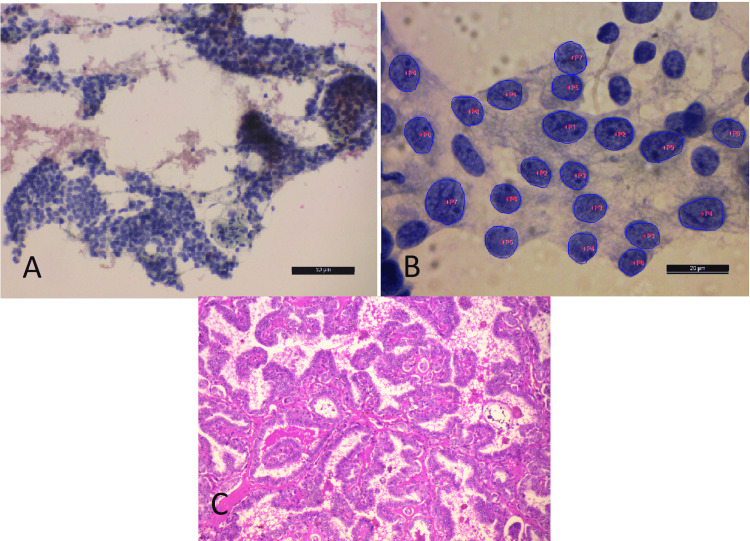
A) Epithelial cell clusters in case of papillary carcinoma (PAP x10). B) Corresponding software morphometric image with labeled nuclei (x1000). C) Corresponding histology showing features of papillary carcinoma of the thyroid (H&E x10) PAP - Papanicolaou

**Figure 4:**
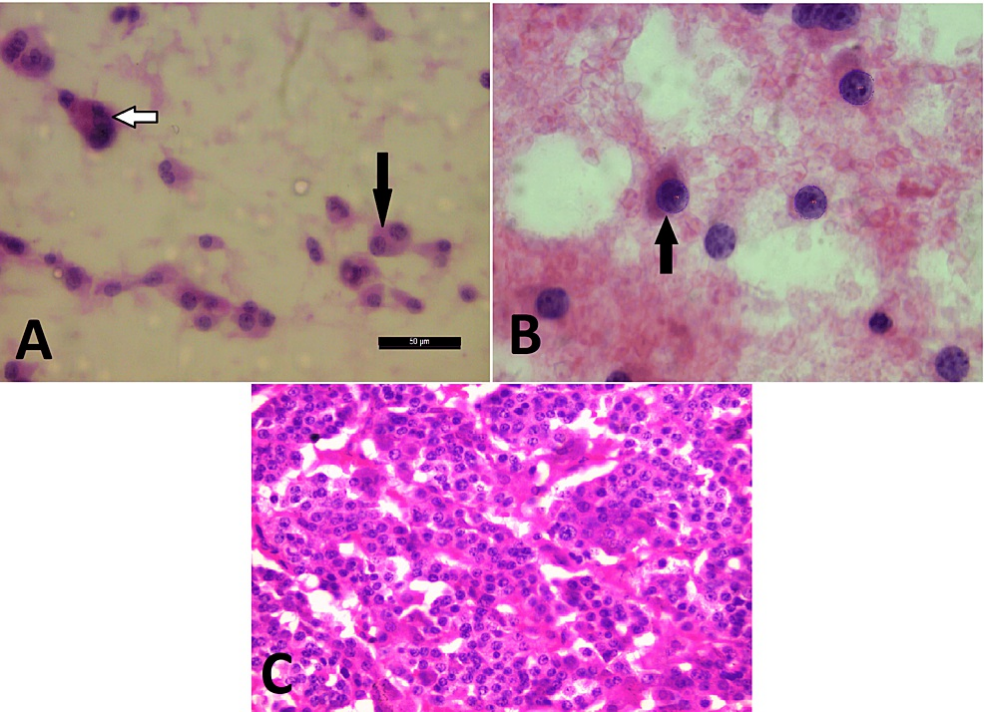
A) Scattered binucleated and single nucleated cells with plasmacytoid morphology having eccentric nucleus (black arrows) and a multinucleated cell (white arrow) in a case of medullary carcinoma of the thyroid (H&E x40). B) Software image showing plasmacytoid cells with a labeled nucleus (arrow) (x1000). C) Corresponding histology was confirmatory of the medullary carcinoma of thyroid (H&E x40).

**Figure 5:**
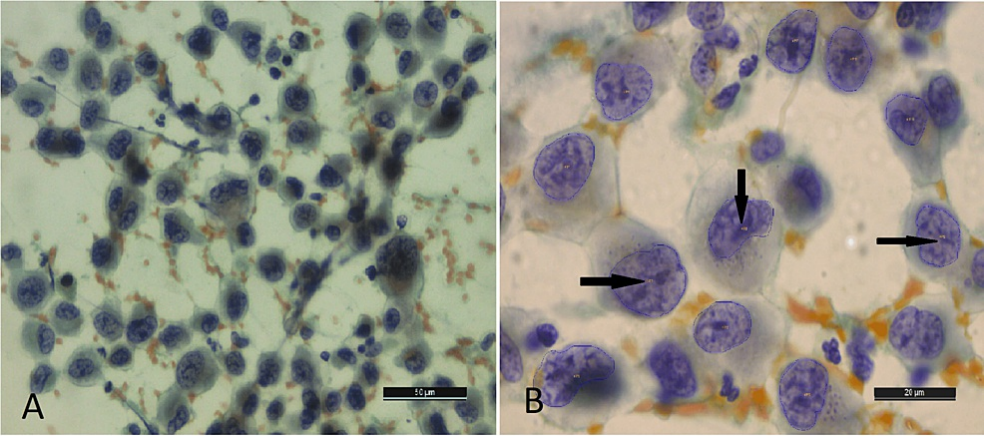
A) Anaplastic cells with marked pleomorphism, irregular nuclear membrane, and coarse chromatin in a case of anaplastic carcinoma of the thyroid (PAP x40). B) Corresponding software image showing anaplastic cells with labeled nuclei (arrows) (x1000) PAP - Papanicolaou

Among the malignant category (N=15) subjected to cytomorphometric analysis (Table 1), papillary carcinoma (10; 66%) was the predominant type which included six cases of conventional papillary carcinoma (PC) (Figure 3) and four cases of follicular variant of papillary carcinoma (FVPC), followed by two cases (13%) each of medullary carcinoma (Figure 4) and anaplastic carcinoma (Figure 5) and one (6%) case of poorly differentiated carcinoma (Table 1).

**Figure 6:**
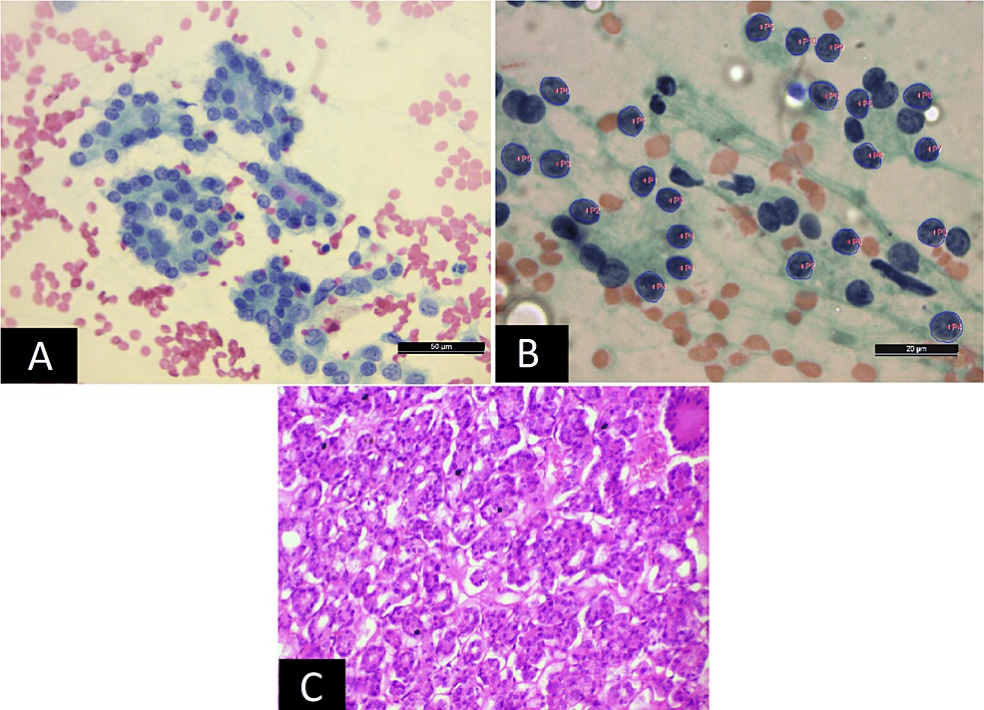
A) Microfollicles in a case categorized as FN/SFN (PAP x40). B) Morphometric software image of the same case showing labeled microfollicular cells (x1000). C) Corresponding histology was of follicular adenoma (H&E x10) The morphometric results of the case were in concordance with the histopathological diagnosis of follicular adenoma. FN/SNF - follicular neoplasm/ suspicious of follicular neoplasm, PAP - Papanicolaou

**Figure 7:**
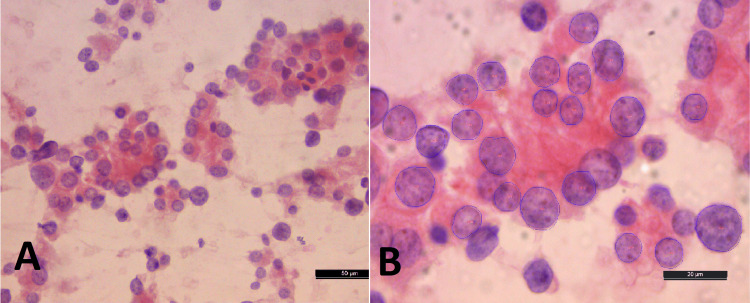
A) Thyroid follicular cells in a case categorized as FN/SFN (H&E x40). B) Corresponding morphometric software image of cells with labeled nuclei (x1000) The morphometric results of the case were in concordance with the histopathological diagnosis of follicular carcinoma. FN/SNF - follicular neoplasm/ suspicious of follicular neoplasm, PAP - Papanicolaou

Among the FN/SFN category (N=20), follicular adenoma (FA) (Figure 6) was confirmed in 11 (55%) cases, and follicular carcinoma (FC) (Figure 7) was confirmed in nine (45%) cases (Table 1).

The data was analyzed to obtain mean with standard deviation values for all 14 parameters in the benign, FN/SFN, and malignant categories. The p-value obtained for each parameter was statistically significant (p<0.001) (Table 3). The ANOVA test was also applied to compare means between these three broad categories (Table 3). 

**Table 3:** Application of ANOVA test for comparing means between benign, follicular neoplasm/ suspicious of follicular neoplasm, and malignant categories of The Bethesda System for Reporting Thyroid Cytopathology

Parameters	Benign	Follicular neoplasm/ suspicious of follicular neoplasm	Malignant	ANOVA p-value
	Mean ± SD	Mean ± SD	Mean ± SD	
Aspect ratio	1.1985 ± 0.12	1.16 ± 0.11	1.31 ± 0.22	<0.001
Intensity (mean)	141.83 ± 21.30	87.47 ± 11.52	111.86 ± 19.93	<0.001
Diameter (mean)	115.22 ± 16.40	125.38 ± 16.17	170.55 ± 44.60	<0.001
Perimeter (polygonal)	373.21 ± 51.43	406.36 ± 54.09	568.74 ± 157.02	<0.001
Roundness	1.05 ± 0.02	1.05 ± 0.11	1.10 ± 0.07	<0.001
Area (polygonal)	10705.45 ± 3056.26	12620.24 ± 3255.50	24848.63 ± 13968.52	<0.001
Fractal dimension	1.04 ± 0.01	1.03 ± 0.04	1.02 ± 0.01	<0.001
Feret diameter (Caliper mean)	118.33 ± 16.24	128.61 ± 16.62	179.11 ± 49.21	<0.001
Margination	0.45 ± 0.04	0.43 ± 0.04	0.41 ± 0.02	<0.001
Heterogeneity	0.04 ± 0.03	0.04 ± 0.04	0.06 ± 0.05	<0.001
Clumpiness	0.15 ± 0.13	0.14 ± 0.14	0.29 ± 0.19	<0.001
Circularity	0.79 ± 0.06	0.81 ± 0.07	0.74 ± 0.09	<0.001
Radii (mean)	57.63 ± 8.20	62.71 ± 8.09	85.32 ± 22.31	<0.001
Fournier Description	0.95 ± 0.05	0.95 ± 0.11	1.02 ± 0.09	<0.001

The post hoc Games-Howell multiple comparison test was also applied for comparing combinations of groups such as benign-FN/SFN group, benign-malignant, and malignant-FN/SFN groups (Table 4).

**Table 4:** Post hoc Games-Howell multiple comparison test for comparing combinations of groups constituting benign, follicular neoplasm/ suspicious of follicular neoplasm, and malignant categories FN/SFN - follicular neoplasm/ suspicious of follicular neoplasm

Parameters	ANOVA p-value	Benign-FN/SFN p-value	Benign-malignant p-value	Malignant-FN/SFN p-value
Aspect ratio	<0.001	<0.001	<0.001	<0.001
Intensity (mean)	<0.001	<0.001	0.001	<0.001
Diameter (mean)	<0.001	<0.001	<0.001	<0.001
Perimeter polygon	<0.001	<0.001	0.001	<0.001
Roundness	<0.001	0.999	<0.001	<0.001
Area (Polygonal)	<0.001	<0.001	<0.001	<0.001
Fractal dimension	<0.001	0.002	<0.001	<0.001
Feret Diameter (Caliper mean)	<0.001	<0.001	<0.001	<0.001
Margination	<0.001	<0.001	0.001	<0.001
Heterogeneity	<0.001	0.904	<0.001	<0.001
Clumpiness	<0.001	0.704	<0.001	<0.001
Circularity	<0.001	0.021	<0.001	<0.001
Radii (mean)	<0.001	<0.001	<0.001	<0.001
Fourier description	<0.001	0.832	<0.001	<0.001

Similarly, we analyzed data to obtain mean with standard deviation for each of the 14 parameters and obtained statistically significant p-values (p<0.001) for distinguishing thyroid nodules with predominant follicular patterns, such as follicular variant of papillary (FVPC), follicular adenoma (FA), and follicular carcinoma (FC) (Table 5). The ANOVA test was also applied to compare means between these three categories of thyroid nodules with predominant follicular patterns (Table 5).

**Table 5:** Application of ANOVA test to compare means between thyroid nodules with predominant follicular pattern

Parameters	Follicular variant of papillary carcinoma (FVPC) (N=4)	Follicular adenoma (FA) (N=11)	Follicular carcinoma (FC) (N=9)	ANOVA p-value
	Mean ± SD	Mean ± SD	Mean ± SD	
Aspect ratio	1.26 ± 0.35	1.14 ± 0.08	1.15 ± 0.09	<0.001
Intensity (mean)	127.70 ± 14.44	93.17 ± 10.55	89.46 ± 6.03	<0.001
Diameter (mean)	133.60 ± 13.77	118.87 ± 11.24	127.01 ± 12.37	<0.001
Perimeter (polygon)	437.81 ± 45.11	386.22 ± 44.50	409.74 ± 39.72	<0.001
Roundness	1.09 ± 0.31	1.0696 ± 0.19	1.0497 ± 0.02	0.142
Area (polygonal)	14302.44 ± 2661.12	11243.87 ± 2059.54	12850.18 ± 2580.12	<0.001
Fractal dimension	1.03 ± 0.00	1.04 ± 0.010	1.03 ± 0.00	<0.001
Feret Diameter (Caliper mean)	138.21 ± 14.012	121.70 ± 11.35	129.98 ± 12.51	<0.001
Margination	0.42 ± 0.026	0.45 ± .042	0.42 ± 0.036	<0.001
Heterogeneity	0.04 ± 0.047	0.02 ± 0.02	0.06 ± 0.045	<0.001
Clumpiness	0.16 ± 0.17	0.12 ± 0.14	0.17 ± 0.14	0.034
Circularity	0.76 ± 0.08	0.82 ± 0.05	0.82 ± 0.057	<0.001
Radii (mean)	66.82 ± 6.92	59.4553 ± 5.62015	63.52 ± 6.18	<0.001
Fourier description	0.98 ± 0.081	0.95 ± 0.18	0.93 ± 0.041	<0.001

A post hoc Games-Howell multiple comparison test was also applied for comparing combinations of groups such as FVPC-FA, FVPC-FC, and FA-FC constituted by nodules with predominant follicular patterns (Table 6).

**Table 6:** Post hoc Games-Howell multiple comparison test for comparing combinations of groups constituted by thyroid nodules with predominant follicular patterns FVPC - follicular variant of papillary carcinoma, FA - follicular adenoma, FC - follicular carcinoma

Parameters	p-values			
	ANOVA	FVPC-FA	FVPC-FC	FA-FC
Aspect ratio	<0.001	<0.001	<0.001	<0.814
Intensity (mean)	<0.001	<0.001	<0.001	0.002
Diameter (mean)	<0.001	<0.001	<0.001	<0.001
Perimeter polygon	<0.001	<0.001	0.001	<0.001
Roundness	0.142	0.528	0.025	0.472
Area (polygonal)	<0.001	<0.001	<0.001	<0.001
Fractal dimension	<0.001	<0.001	0.145	<0.001
Feret Diameter (Caliper mean)	<0.001	<0.001	<0.001	<0.001
Margination	<0.001	<0.001	0.987	<0.001
Heterogeneity	<0.001	<0.001	0.001	<0.001
Clumpiness	0.034	0.984	<0.037	<0.047
Circularity	<0.001	<0.001	<0.001	0.941
Radii (mean)	<0.001	<0.001	<0.001	<0.001
Fourier description	<0.001	0.134	<0.001	0.468

## Discussion

Morphometry is an innovative technique with its application previously documented in breast, oral, and salivary gland cytology [4-6]. Although there are studies in literature documenting the application of morphometric analysis on aspirates of thyroid nodules for distinguishing benign and malignant nodules, there are only a few studies with variable results addressing the distinction of FN/SFN of the TBSRTC [7,8]. In our study, we analyzed 14 parameters, including chromatin texture parameters such as clumpiness and heterogeneity, and found statistically significant results (p<0.001) for distinguishing FN/SFN category from benign and malignant categories (Tables 3, 4). Similarly, Razavi et al. also applied nuclear morphometery on aspirates of the FN/SFN category. However, their study measured only seven validated parameters with no significant difference in morphometric measurements for distinguishing benign and malignant from FN/SFN nodules [8]. Yashaswini et al. and Wright et al. also emphasized on the preoperative diagnostic value of morphometry and documented that malignant nodules have larger areas and perimeters as compared to benign nodules, similar to the results of our study [9,10]. Nugroho et al. also reported that parameters such as circularity and aspect ratio could be used to correctly classify the malignancy status of thyroid nodules [11]. In the present study, apart from higher values for mean aspect ratio, diameter, perimeter, area, feret diameter, radii, Fourier description in the malignant category, the chromatin texture parameters such as heterogeneity and clumpiness also showed slightly higher values in the malignant category with statistical significance (p<0.001) as compared to benign and FN/SFN categories (Table 3).

In the present study, a post hoc Games-Howell multiple comparison test was applied for comparing combinations of groups constituted by benign, FN/SFN, and malignant categories (Table 4), which also showed highly significant results (p<0.001) except for a few insignificant results for roundness (p=0.999), heterogeneity (p=0.904) and clumpiness (p=0.704) within comparative group benign-FN/FSN (Table 4).

We obtained statistically significant results (p<0.001) for distinguishing nodules with predominant follicular patterns, such as follicular variant of papillary carcinoma (FVPC), follicular adenoma (FA), and follicular carcinoma (FC) (Table 5). The mean aspect ratio, diameter, perimeter, area, feret diameter, and radii were highest in FVPC, followed by FC and FA, respectively. Also, the cells of FVPC were found to be less circular as compared to both FC and FA (Table 5). Similar results were obtained by Aiad et al., who also studied the morphometric parameters of thyroid nodules with predominant follicular patterns and documented that the majority of nuclear parameters were significantly higher in FVPC than in FA or FC [12]. Rout et al. also showed that nuclear area and mean nuclear diameter were highest in papillary carcinoma than in FC and FA [13]. However, Aiad et al. and Karslioğlu et al. documented that quantitative nuclear assessment did not yield significant results in differentiating FC from FA because of the considerable overlap of nuclear morphometric parameters [12,14]. In contrast to these studies, our results distinguished FA and FC by statistically significant p-values (p<0.001) for the majority of parameters measured (Table 5). The parameters such as mean aspect ratio, diameter, perimeter, area, feret diameter, and radii had higher values in FC as compared to FA (Table 5). Ciobanu et al. also reported that the nuclear area and mean nuclear diameter in follicular carcinoma were significantly larger than follicular adenoma [15]. An additional advantage of the present study is the measurement and analysis of chromatin texture parameters, such as heterogeneity and clumpiness, apart from conventional nuclear parameters (Table 5). The chromatin texture parameter, such as heterogeneity and clumpiness, could distinguish FVPC, FA, and FC, as these parameters were highest in FC, followed by FVPC, and least in FA (Table 5). The application of the post hoc Games-Howell test for comparison of combinations of groups between FVP, FA, and FC (Table 6) also showed significant p-values (p<0.001) except for insignificant results for aspect ratio (p=0.814), roundness (p=0.472), circularity (p 0.941) and Fourier description (p=0.468) in FA-FC comparative group and insignificant p-value for roundness (p=0.528) and circularity (p=0.984) in FVP-FA comparative group (Table 6).

Limitations of the study

The study included a total of 50 cases for cytomorphometric image analysis. Although the sample size is small, the results of the study can contribute to larger studies with large sample sizes in the near future to further affirm the diagnostic role of cytomorphometry in thyroid cytopathology.

## Conclusions

The results of our study reveal that the cytomorphometric image analysis combined with cytomorphology has the potential for distinguishing not only the benign, FN/SFN, malignant thyroid nodules of TBSRTC but can also differentiate nodules with predominant follicular patterns, such as follicular variant of papillary carcinoma, follicular adenoma, and follicular carcinoma. The results also arouse hope and can contribute to larger studies with larger sample sizes in the near future addressing the subject and affirming the diagnostic role of cytomorphometric image analysis in the cytopathology of thyroid nodules.

## References

[1] Cibas ES, Ali SZ (2017). The 2017 Bethesda System for Reporting Thyroid Cytopathology. Thyroid.

[2] Baloch ZW, Fleisher S, LiVolsi VA, Gupta PK (2002). Diagnosis of "follicular neoplasm": a gray zone in thyroid fine-needle aspiration cytology. Diagn Cytopathol.

[3] Bongiovanni M, Bellevicine C, Troncone G, Sykiotis GP (2019). Approach to cytological indeterminate thyroid nodules. Gland Surg.

[4] Jannat HE, Saleh AF, Hossain SA, Das SR, Hossain T (2022). Role of nuclear morphometry in the cytologic evaluation of benign and malignant breast lesions. Mymensingh Med J.

[5] Oz ZS, Bektas S, Battal F, Atmaca H, Ermis B (2014). Nuclear morphometric and morphological analysis of exfoliated buccal and tongue dorsum cells in type-1 diabetic patients. J Cytol.

[6] Chaurasia JK, Gupta V, Mayank V, Tiwari IR, Joshi D, Goel G, Kapoor N (2020). Role of nuclear morphometry in diagnosis of salivary gland neoplasms. Diagn Cytopathol.

[7] Razavi MA, Wong J, Akkera M (2020). Nuclear morphometry in indeterminate thyroid nodules. Gland Surg.

[8] Khatri P, Choudhury M, Jain M, Thomas S (2017). Role of morphometry in the cytological differentiation of benign and malignant thyroid lesions. J Cytol.

[9] Yashaswini R, Suresh TN, Sagayaraj A (2017). Cytological evaluation of thyroid lesions by nuclear morphology and nuclear morphometry. J Cytol.

[10] Wright RG, Castles H, Mortimer RH (1987). Morphometric analysis of thyroid cell aspirates. J Clin Pathol.

[11] Nugroho HA, Frannita EL, Ardiyanto I (2019). Computer aided diagnosis for thyroid cancer system based on internal and external characteristics. J King Saud Univ Comput Inform Sci.

[12] Aiad H, Abdou A, Bashandy M, Said A, Ezz-Elarab S, Zahran A (2009). Computerized nuclear morphometry in the diagnosis of thyroid lesions with predominant follicular pattern. Ecancermedicalscience.

[13] Rout P, Shariff S (1999). Diagnostic value of qualitative and quantitative variables in thyroid lesions. Cytopathology.

[14] Karslioğlu Y, Celasun B, Günhan O (2005). Contribution of morphometry in the differential diagnosis of fine-needle thyroid aspirates. Cytometry B Clin Cytom.

[15] Ciobanu D, Căruntu ID, Vulpoi C, Florea N, Giuşcă SE (2006). Morphometric parameters and silver stain used in diagnosis of thyroid follicular diseases. Rom J Morphol Embryol.

